# GOING MOBILE: ADAPTING LATE-LIFE PSYCHOTHERAPIES FOR MOBILE DEVICES

**DOI:** 10.1093/geroni/igae098.1845

**Published:** 2024-12-31

**Authors:** Felipe Jain, Paulina Gutierrez-Ramirez, Maria Melero-Dominguez, Miranda Zea, Liliana Ramirez Gomez

**Affiliations:** Depression Clinical and Research Program / Massachusetts General Hospital, Boston, Massachusetts, United States; Massachusetts General Hospital, Boston, Massachusetts, United States; Massachusetts General Hospital, Boston, Massachusetts, United States; Massachusetts General Hospital, Boston, Massachusetts, United States; Massachusetts General Hospital, Boston, Massachusetts, United States

## Abstract

Smartphone psychotherapy applications have the potential for broad scalability, objective treatment compliance monitoring, and passive monitoring of symptom changes (e.g. by leveraging smartphone sensors such as accelerometer). The majority of older adults own and utilize smartphones, but older adults are underrepresented in smartphone research. Moreover, considerations for adapting psychotherapy information for older adults to the smartphones are not well-described. Older adult family caregivers of persons living with dementia (PlD) describe numerous barriers to participation in in-person therapies including travel time and caregiver burden. Mentalizing imagery therapy is a guided imagery and mindfulness approach that has been shown in in-person studies in family PlD caregivers to reduce depression and anxiety, and to strengthen cognitive control (dorsolateral prefrontal) brain connectivity. We present a case study of the adaptation of mentalizing imagery therapy and caregiver skills training for family caregivers of PlD to a mobile application supported by optional virtual groups. We describe user-centered participatory design of the mobile and virtual therapy application including mobile architecture, data storage and security, feature development, collection of passive accelerometer data, embedded chat group, and development timeline. Initial feasibility results are presented from forty older adult family caregivers of PlD, including retention, compliance, system usability, and usage information. These results demonstrate meeting pre-specified feasibility benchmarks and describe important challenges with adaptations of psychotherapy to smartphone. We also demonstrate feasibility of collecting continuous smartphone accelerometer data, and preliminary results from a machine learning data analytic pipeline that achieved >85% accuracy for estimation of weekly mood ratings.

